# Investigation of Serum Ferritin for the Prediction of COVID-19 Severity and Mortality: A Cross-Sectional Study

**DOI:** 10.7759/cureus.31982

**Published:** 2022-11-28

**Authors:** Jihad M Hadi, Hawar M Mohammad, Ako Y Ahmed, Shko S Tofiq, Las B Abdalrahman, Awin A Qasm, Ali M Ameer

**Affiliations:** 1 Medical Laboratory of Science, College of Health Sciences, University of Human Development, Sulaymaniyah, IRQ; 2 Nursing Department, College of Nursing, University of Human Development, Sulaymaniyah, IRQ

**Keywords:** inflammatory markers, serum ferritin, sars-cov-2, coronavirus, covid-19

## Abstract

Background: Acute respiratory failure develops quickly in patients with a severe form of coronavirus disease (COVID-19), caused by the severe acute respiratory syndrome Coronavirus 2 (SARS‑CoV‑2). Despite being commonly acknowledged as a measure of the body's overall iron storage, ferritin's predictive value is associated with COVID-19.

Objective: This study aimed to evaluate the relationship between COVID-19 and serum ferritin levels as the biochemical markers of SARS-CoV-2 infection in Sulaymaniyah, Iraq.

Method: A biochemical test was performed at Baxshin Hospital in the period from February 2022 to April 2022. It was performed on a total of 85 patients (63.53% males and 36.47% females), ranging in age from 25 to 79 years old, with an average age of 48.4 years old. The patient’s blood samples were taken to screen for ferritin levels.

Result: The resulting outcome of this work is high serum ferritin levels for the majority of infected patients. Overall, there is a significant difference between male and female serum ferritin observed with a p-value < 0.05. The median interquartile range (IQR) of serum ferritin was 896 ng/mL for males, while it was only 611 ng/mL for females. The current study showed that age level has a great effect on elevated ferritin levels. It has been discovered that gender impacts increasing ferritin levels; 62% were found to be men and 38% were found to be women, with average ferritin levels of 1111 ng/mL and 712.8 ng/mL, respectively.

Conclusion: SARS-CoV-2 infection causes significant laboratory abnormalities, including a high level of serum ferritin.

## Introduction

A new infectious disease known as severe acute respiratory syndrome (SARS) occurred in the Guangdong province of southern China in 2002. It is mainly characterized by flu-like symptoms, including high fevers exceeding 38 °C, non-productive dyspnea, lymphopenia, and infiltration on chest radiography. In most cases, the resulting pneumonia led to acute breathing problems requiring artificial respirators for the patients [1]. The World Health Organization designated Coronavirus disease-19 (COVID-19) as a contagious infection caused by severe acute respiratory syndrome 2 (SARS-CoV-2) or new Coronavirus (2019-novel) virus (COVID-19). The first occurrence was detected in Wuhan, Hubei Province, China, in December 2019. COVID-19 is a multi-organ dysfunction disease with symptoms such as fever, dry cough, tiredness, breathing problems, and loss of taste and smell. In general, the illness will produce moderate pneumonia in 79% of instances, more severe symptoms such as hypoxia and dyspnea in 15% of cases, and critical and life-threatening circumstances such as respiratory failure, multi-organ dysfunction, and shock in 15% of cases. The majority of instances are asymptomatic, but some are symptomatic, such as those in the elderly or those with a weakened immune system [2,3]. The focus of scientific research was on establishing appropriate therapy regimens to battle the virus as the pandemic's devastating impacts progressed. Nevertheless, early risk stratification techniques and biomarkers were desperately needed to forecast disease progression and identify high-risk people early in the infection. This can help the management achieve the objectives and overcome the scarcity of medical and material resources that was evident throughout this worldwide crisis [4,5].

The inflammatory process caused by the SARS-CoV2 infection likely played a key role in the development of multi-organ damage and the COVID-19 patients' severe outcomes. Early detection of COVID-19 patients with poor prognostic characteristics might be beneficial in terms of a care plan, limiting severe complications and mortality. White blood cell count, lactate dehydrogenase, C-reactive protein, fibrinogen, and D-dimer are all inflammatory indicators that are routinely used in clinical practice to monitor sepsis [6,7]. Iron metabolism has been linked to a number of pathogenetic disease pathways, including infections and a variety of hematological and immunological illnesses [8]. According to recent data published in the literature, iron metabolism can undergo considerable changes, which can be used to predict death in patients admitted to intensive care units. Serum ferritin has also recently been identified as one of the predictors of death in COVID-19 patients [9]. Serum ferritin is a kind of iron storage protein that regulates cellular oxygen metabolism. H and L are the two subunits that make up ferritin. According to previous research, H-ferritin operates as an immune modulator with both pro-inflammatory and immunosuppressive activities. Increased ferritin levels may signal a severe inflammatory reaction in response to viral entrance into the human body and its influence on iron metabolism [10]. Eloseily et al. [11] have reported that an increased ferritin measurement (e.g., >700 ng/mL) should alert clinicians to conduct further diagnostic work-up so that treatment methods can be addressed without delay. Early detection and treatment of cause-specific survival (CSS) have been found to enhance patient outcomes. As a result, we chose to explore serum ferritin as an inflammatory measure in COVID-19 patients. The objective of the current research is to assess whether serum ferritin levels can act as a predictor of severity and mortality in patients with COVID-19 in Sulaymaniyah city, Kurdistan region of Iraq.

## Materials and methods

Materials

All the materials used are needles, tourniquets, cotton, sanitizer, and plaster. Then the samples were taken at Baxshin Hospital. Also, in the procedure of testing, a variety of equipment and instruments have been used, including a sample rack, test tube, real-time PCR, and Cobas-e411. The study was conducted according to the guidelines of the Declaration of Helsinki and approved by the Ethics Committee of the Baxshin Research Center at Baxshin Hospital, approved code (BRC26012022).

Data collection

In this study, from February 2022 to April 2022, 85 serum samples were obtained from COVID-19-infected individuals in the Baxshin hospital in Sulaymaniyah province. For all samples, the COVID-19 test (RT-PCR) was used. In this analysis, several factors, including gender, age, and serum ferritin, were considered in accordance with scientific research ethics to compare the biochemical parameters.

Laboratory testing determination of serum ferritin

First of all, 85 serum samples were collected from all the patients to detect the level of ferritin using an automated (Cobas e411) device (Roche, Germany) with a reference range of fewer than 30-400 ng/mL in men and 13-150 ng/mL in women.

## Results

In this work, all patients were between 28 and 70 years old, with an average age of 48.4 years. The COVID-19 patient group included 85 patients with fixed symptoms such as fever, cough, weakness in the breath (difficulty breathing), and numerous organ dysfunctions. The gender distribution of patients in this research is 54 (63.5%) males and 31 (36.5%) females. Table 1 revealed highly significant findings concerning the relationship of SARS-CoV-2 infection with serum ferritin. Of the 85 patients, 20 deaths were recorded, with a gender distribution of 13 (65%) males and 7 (35%) females. Among the death cases, all the patients had elevated ferritin levels, ranging from 452 to 2000 ng/mL with an average value of 1402 ng/mL. It can be observed that the elevated ferritin levels are high compared to normal levels for the whole patient, as shown in Table 1. Furthermore, normal levels of ferritin were observed in 23 patients, including 15 males and 8 females. The average ferritin levels were found to be 260.5 and 91.27 ng/mL, respectively.

**Table 1:** The relation of serum (ferritin) with COVID-19 infection for 85 patients, including age, and gender.

Statements	Patients no.	% (n=85)	Average ferritin levels (ng/mL)
Age range			
25–35	16	18.82	342.8
36–46	24	28.24	431.6
47–57	26	30.59	913.1
58–68	14	16.47	973.5
69–79	5	5.88	1756.4
Gender			
Male	54	63.53	854
Female	31	36.47	541
Total	85	-	-
P-value = 0.03<0.05			
Normal			
Male (30–400 ng/mL)	15	17.64	260.5
Female (13–150 ng/mL)	8	9.41	91.27
Total	23	27.05	-
Decreased			
Male	1	1.17	19.6
Female	0	0	0
Total	1	1.17	-
Increased			
Male	38	44.7	1111
Female	23	27	712.8
Total	61	71.7	911.9
Dead			
Male	13	65	1328
Female	7	35	1540
Total	20	-	1402.5

**Figure 1:**
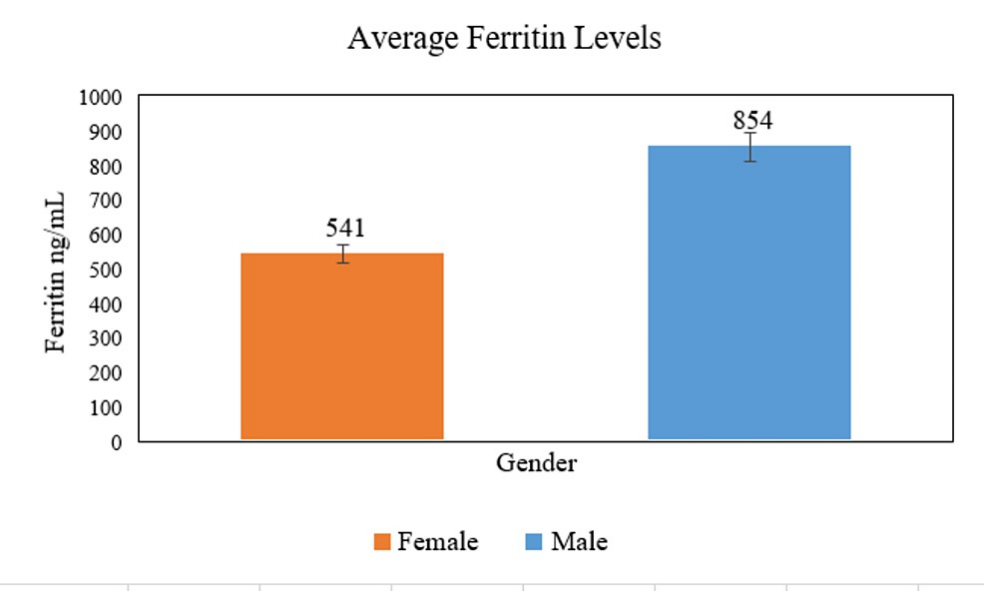
The variation of ferritin levels along with gender for the whole 85 patients.

It is worth mentioning that the average ferritin levels are different for both genders. The serum ferritin level for male patients is significantly higher than that for female patients, where it is found to be 854 ng/mL in males while it is only 541 ng/mL in female patients, as illustrated in Figure 1.

**Figure 2:**
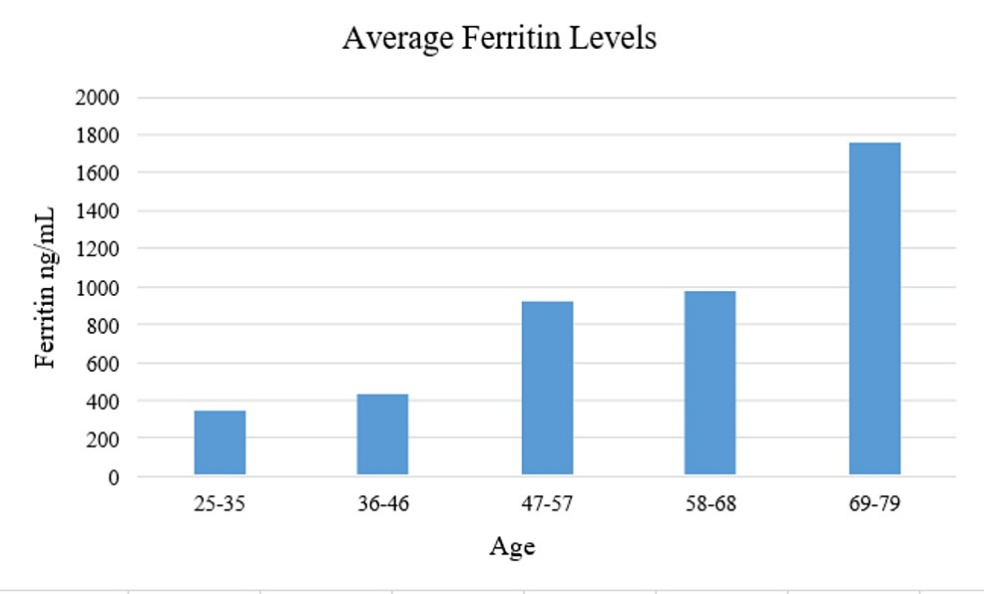
The level of ferritin in the patients according to the age.

On the other hand, according to the study, 61 patients had ferritin levels higher than the normal range. The increased ferritin level was displayed at 62% in males and 38% in females. Furthermore, patients who have died of COVID-19 infection with ages ranging from 38 to 78 years showed high ferritin levels ranging from 575 to 2000 ng/mL. Figure 2 demonstrates the ferritin levels according to age level. It can be seen that the ferritin level increased with the increment in age. The highest average ferritin level of 1756 ng/mL was observed for the patients in the age group of 69-79 years old. Whereas, the value of the average ferritin level was found to be 342.8 ng/mL for the minimum age group. Table 2 represents the two-sample assuming unequal variances by gender. It is noticeable that the median interquartile range (IQR) for males has been found to be 896 ng/mL, while the female patients showed a lower value of 611 ng/mL. Furthermore, the probability p-value is lower than the alpha (p<0.05), which means there is a significant difference between genders in their ferritin levels.

**Table 2:** Two-sample assuming unequal variances according to gender.

Serum ferritin	Male	Female
Mean	854.6	541.4
Observations	54	31
Median interquartile range	896.3	611.8
Hypothesized mean difference	0	
t-stat	2.12	
P (T≤t) one-tail	0.019	
t-critical one-tail	1.68	
P (T≤t) two-tail	0.038	
t-critical two-tail	1.99	

If the t-stat is larger than the original two-tail value, the H0 is rejected. However, since 2.11 is smaller than 1.99, the null hypothesis (H0) has been rejected, and the data support the alternative hypothesis (H1). Thus, the ferritin level of males and females cannot be considered equal. Table 2 showed that there was a mean difference in male and female ferritin levels, with a P-value < 0.05 showing that there is a significant difference between male and female serum ferritin.

## Discussion

Systemic inflammatory response syndrome (SIRS), sepsis, and severe sepsis are the stages of inflammation that occur when infection arises [12]. Early detection of urgent illnesses and risk-based management would help to relieve the pressure on limited medical resources and could lower mortality. Low platelet-to-lymphocyte ratios [13], thrombocytopenia [12], and low lymphocyte-to-C-reactive protein ratios [14] have all been linked to severe disease in new findings.

Although ferritin is listed as an acute-phase protein, there is a lack of research providing the specific modified levels, which results in uncertainty over its interpretation [15]. Recently, the importance of IL-6 in COVID patients has increased, and the traditional sepsis score parameters do not fully explain or provide answers to all of the concerns raised by the cytokine storm. Although iron parameters are not yet regarded as standard biomarkers to track septic development, the relationship between IL-6 and iron metabolism is well recognized [16]. Ferritin, transferrin, or other iron parameters aren't even mentioned in the most recent COVID recommendations (COVID-19) [17].

As stated in this study, the inflammatory process we are searching for appears differently in COVID patients than in typical acute inflammation, such as emergency surgery. The acute inflammatory process corresponds with the standard measures but not with ferritin modification in patients admitted for acute abdominal or other surgical pathologies. Contrarily, iron modification appears to happen right away in COVID individuals. Only 4 of the 16 trials were examined in a meta-analysis by Zeng et al. [18], however, he stressed that ferritin levels might categorize the severity of COVID patients.

While the number of Coronavirus disease 2019 (COVID‐19) cases is increasing day by day, there is limited information known about the hematological and laboratory findings of the disease. We aimed to investigate whether a serum ferritin level predicts mortality is a marker for rapid progression in inpatients. The study included 20 dead patients due to being infected by COVID‐19 and 65 patients who were hospitalized and recovered as the control group. Some of the cases displayed no effect on ferritin levels. The same facts and results were found by many researchers, such as Vargas-Vargas and Cortés-Rojo [8] and Gómez-Pastora et al. [19]. According to their research, lab discoveries in patients with acute COVID-19 displayed data consistent with a cytokine storm, including raised inflammatory markers and ferritin, which has been associated with crucial and life-threatening sickness.

On the one hand, 23 patients (27%) displayed normal levels of ferritin. On the other hand, COVID-19 had a significant effect on ferritin levels for a majority of patients. Ferritin levels have been high compared to the normal range, and patients were also treated. The abnormal ferritin levels were found in 12 patients, ranging from 500 to 1928 ng/mL, including two women.

Furthermore, the resulting outcome of this work showed that ferritin levels increased with the increment in age. This could be due to the chronic diseases of some of the oldest patients. Thus, it can be said that the age level has a great impact on the ferritin level as well as the mortality of the patients. In addition, according to Zacharski et al. [20], serum ferritin levels decreased with increasing age in control patients, apparently because patients with higher iron stores were more likely to die earlier. Second, younger iron reduction patients were more likely to comply with the phlebotomy intervention. The function of the serum ferritin level as a predictive biochemical marker in the detection of COVID-19 disease is of great importance since ferritin is strongly linked to viral multi-organ damage and elevated inflammatory cytokines. This fact was proved by Habib et al. [21].

It is well known that a ferritin level higher than the expected normal range may also be a sign of hyperthyroidism, liver illness, rheumatoid arthritis, drug toxicity, or other inflammatory diseases.

This study has significant limitations, including the small number of patients included, which was mostly owing to atypical serum ferritin tests during the beginning period, as well as its retroactive nature. Thus, the findings of our investigation must be validated in a larger population study. Also, because the data were generated clinically rather than methodically, we did not include other iron-related indicators such as serum iron and transferrin. Furthermore, due to limited medical resources, most patients did not have sputum testing for pathogenic bacterial or fungal identification during their hospitalization. Therefore, serum ferritin levels should be interpreted very carefully in the context of other co-morbid conditions/diseases. As a result, a broader analysis would be required to confirm our findings.

## Conclusions

Even though ferritin is regarded as an acute-phase protein, its function in observing inflammation is still unclear, and as a result, it is not frequently used. Therefore, without a precise interpretation, the function of iron metabolism in COVID infection is increasing, and the study appears to support this finding. Serum ferritin levels could be regarded as preliminary assessments since they are simple to test (such as indecisiveness in an emergency) and because they assist medical professionals in identifying COVID-19. Moreover, our finding raises the possibility of using ferritin levels as a sign of COVID infection.
